# The complete mitochondrial genome of the enigmatic bigheaded turtle (*Platysternon*): description of unusual genomic features and the reconciliation of phylogenetic hypotheses based on mitochondrial and nuclear DNA

**DOI:** 10.1186/1471-2148-6-11

**Published:** 2006-02-07

**Authors:** James F Parham, Chris R Feldman, Jeffrey L Boore

**Affiliations:** 1 Department of Evolutionary Genomics, DOE Joint Genome Institute and Lawrence Berkeley National Laboratory, 2800 Mitchell Drive, Walnut Creek, CA, 94598, USA; 2 Museum of Paleontology, University of California, Berkeley, CA, 94720, USA; 3 Department of Biology, Utah State University, Logan, UT, 84322, USA; 4 Department of Integrative Biology, 3060 Valley Life Science Building, University of California, Berkeley, CA, 94720, USA

## Abstract

**Background:**

The big-headed turtle (*Platysternon megacephalum*) from east Asia is the sole living representative of a poorly-studied turtle lineage (Platysternidae). It has no close living relatives, and its phylogenetic position within turtles is one of the outstanding controversies in turtle systematics. *Platysternon *was traditionally considered to be close to snapping turtles (Chelydridae) based on some studies of its morphology and mitochondrial (mt) DNA, however, other studies of morphology and nuclear (nu) DNA do not support that hypothesis.

**Results:**

We sequenced the complete mt genome of *Platysternon *and the nearly complete mt genomes of two other relevant turtles and compared them to turtle mt genomes from the literature to form the largest molecular dataset used to date to address this issue. The resulting phylogeny robustly rejects the placement of *Platysternon *with Chelydridae, but instead shows that it is a member of the Testudinoidea, a diverse, nearly globally-distributed group that includes pond turtles and tortoises. We also discovered that *Platysternon *mtDNA has large-scale gene rearrangements and possesses two, nearly identical, control regions, features that distinguish it from all other studied turtles.

**Conclusion:**

Our study robustly determines the phylogenetic placement of *Platysternon *and provides a well-resolved outline of major turtle lineages, while demonstrating the significantly greater resolving power of comparing large amounts of mt sequence over that of short fragments. Earlier phylogenies placing *Platysternon *with chelydrids required a temporal gap in the fossil record that is now unnecessary. The duplicated control regions and gene rearrangements of the *Platysternon *mtDNA probably resulted from the duplication of part of the genome and then the subsequent loss of redundant genes. Although it is possible that having two control regions may provide some advantage, explaining why the control regions would be maintained while some of the duplicated genes were eroded, examples of this are rare. So far, duplicated control regions have been reported for mt genomes from just 12 clades of metazoans, including *Platysternon*.

## Background

**Figure 1:**
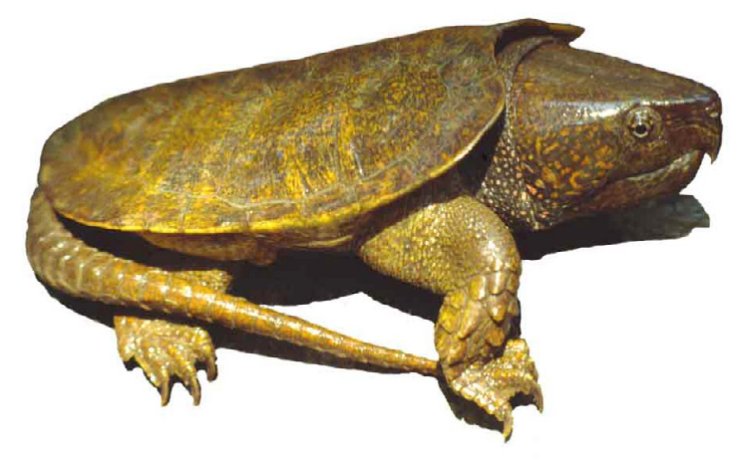
**The Asian big-headed turtle (*Platysternon*)**. A live *Platysternon *showing the characteristic large head and long tail.

Molecular studies have made significant contributions to our understanding of higher-level turtle evolutionary relationships [1-3], but there are still some areas of uncertainty or apparent conflict between data sets. One of the major outstanding issues is the placement of the enigmatic "big-headed turtle" of Asia (*Platysternon megacephalum*; Fig. 1). *Platysternon megacephalum *is the sole living representative of a poorly-studied turtle lineage (Platysternidae), and its phylogenetic position within turtles is not easily established. It ranges from Myanmar, Thailand, Laos, and Vietnam to southern China where it inhabits rocky mountain streams. *Platysternon *feeds on a variety of prey, including freshwater crustaceans and molluscs. To effect this durophagous diet, *Platysternon *has evolved powerful jaw muscles and a correspondingly hypertrophied cranium. In addition to its large head, it also has an unusually long tail for a turtle.

**Figure 2:**
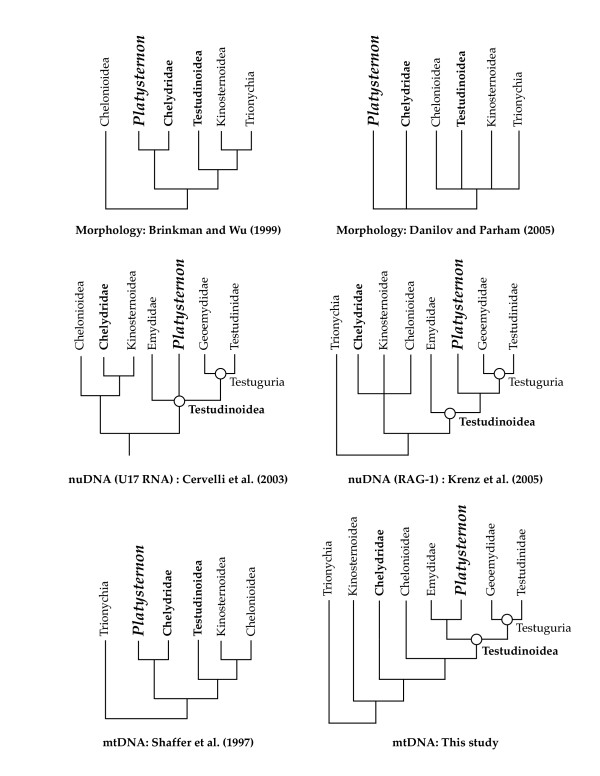
**Hypotheses for *Platysternon *relationships**. Examples of phylogenetic hypotheses proposed for *Platysternon *based on morphology [4, 23], small mtDNA sequences (fragments of *cob *and *rrnS *combined) [1], nuDNA (U17 snoRNA, RAG-1) [2, 3], and large mtDNA sequences [this study].

Two hypotheses are the strongest contenders for the phylogenetic position of *Platysternon*, with proponents of each position coming from molecular and morphological systematists. Based on some studies of its morphology [1,4,5] and mitochondrial DNA [1], *Platysternon *has been phylogenetically linked to New World snapping turtles (Chelydridae; Fig. 2). Indeed, *Platysternon *and chelydrids (two extant species) are superficially similar since both have large heads and long tails. However, other morphological comparisons [6-8] and studies of serology [9] have supported a relationship to the more diverse (~150 extant species) group that includes pond turtles and tortoises (Testudinoidea). Testudinoids are found on all continents except Australia and Antarctica, but are particularly diverse in Asia and North America.

Multiple studies have differed in the placement of *Platysternon*, with the results contrasted in Figure 2. Recent studies of the phylogenetic position of *Platysternon *using nuDNA (RAG-1 and U17 snoRNA) strongly supported testudinoid affinities [2,3]. One of these studies [3] gave a detailed review of the conflicting signals from other data sets (mtDNA and morphology). These authors acknowledged the dissenting voices on the "*Platysternon *as a chelydrid" scheme from morphologists, but it should be noted that all such morphological hypotheses were not proposed in an explicit cladistic framework. Meanwhile, the most recent cladistic analysis of osteological characters [10] could not resolve the position of *Platysternon *beyond placing it in the same major clade (Cryptodira) that includes most extant turtle lineages including the testudinoids and chelydrids, but also softshell turtles (Trionychia), mud turtles (Kinosternoidea), and sea turtles (Chelonioidea). The combined phylogenetic analysis of short sequences of mtDNA (fragments of *cob *and *rrnS*) placed *Platysternon *next to chelydrids [1].

In order to pursue a definitive resolution of this issue, we sequenced the complete mt genome of *Platysternon *and nearly complete mt genome of a chelydrid and kinosternoid and compared these data to mt genomes published for other turtle lineages. In the process, we discovered several unusual mt genomic features that further distinguish this enigmatic turtle. We describe these genomic features and review the phylogenetic position of *Platysternon*.

## Results and discussion

### Phylogenetic position of *Platysternon*

**Figure 3:**
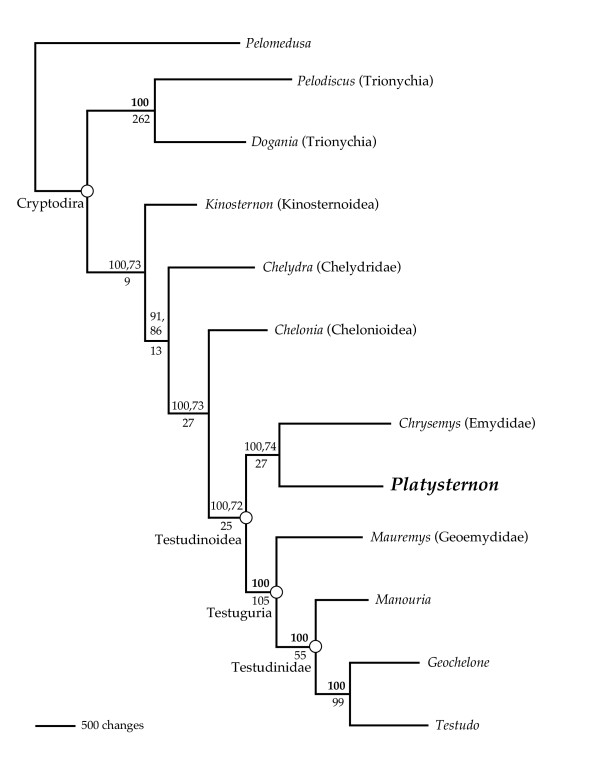
**Phylogenetic relationships of turtles based on large mt alignments**. Parsimony phylogram of the single tree recovered by all analyses (MP, BI, ML). Numbers above branches refer to BI posterior probabilities and MP bootstraps respectively, while a single bold number above a node indicates the identical BI and MP support for that node. Numbers below the nodes refer to decay indices.

**Figure 4:**
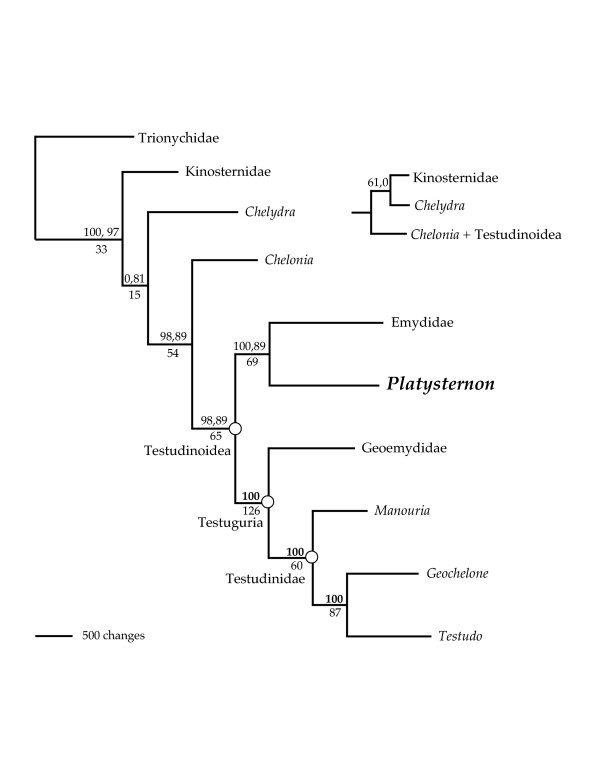
**Phylogenetic relationships of cryptodires based on a combined analysis of large mt sequences and nuDNA**. Parsimony phylogram. Inset: alternative topology recovered by the BI analysis. Numbers above branches refer to BI posterior probabilities and MP bootstraps respectively, while a single bold number above a node indicates the identical BI and MP support for that node. Numbers below the nodes refer to decay indices.

Our phylogenetic analyses of 7.2–16.2 kilobases (kb) of mtDNA for 12 turtles (>182 kb total) using maximum parsimony (MP, L = 19481), Bayesian inference (BI, harmonic mean -lnL = 94787.18), and maximum likelihood (ML, -lnL = 95683.6880) methods place *Platysternon *within Testudinoidea (Fig. 2, 3). Although the MP bootstrap values for testudinoid affinities are not strong, the traditional hypothesis linking *Platysternon *with Chelydridae was rejected by statistical tests of hypothesis compatibility (MP, Wilcoxon signed ranks test: L difference = 68, z = -2.2489, p = 0.0245; ML, SH test: -lnL difference = 38.5531, p = 0.0336). Although our tree agrees with the nuDNA [2,3] in refuting an affinity to Chelydrids and placing *Platysternon *firmly within Testudinoidea, our results differ by weakly placing *Platysternon *as sister to the Emydidae rather than sister to Testuguria. While MP constraint searches that retained only those trees wherein *Platysternon *is sister to the Testuguria are significantly longer than the unconstrained estimate of turtle phylogeny (Wilcoxon signed ranks test: L difference = 62, z = -2.0769, p < 0.0001), identical ML constraint searches failed to produce topologies that were significantly worse solutions than the unconstrained ML tree (SH test: 15.4560, p = 0.264). Furthermore, the placement of *Platysternon *with Testuguria received weak nodal support in the nuDNA studies (52 or <50 MP bootstrap) [2,3] so the difference here is not seen as an important conflict between mtDNA and nuDNA. Other conflicts between the mtDNA and nuDNA involve the outgroups of Testudinoidea within Cryptodira, though both agree that Trionychia is the most basal cryptodiran clade [1,3] (Fig. 2). Where the nuDNA phylogenies differ from our tree, the nodal support in the nuDNA studies is either weak (<50 MP bootstrap) or else the topology differs depending on which phylogenetic method of searching was used. As would be expected, a combined analysis (MP, L = 21819; BI, harmonic mean -lnL = 103,332.71) can not resolve these conflicts (Fig. 4). Additional large mtDNA sequences as well as those from additional nuDNA markers may help resolve these discrepancies.

Our phylogeny reconciles the previous conflict between mtDNA and nuDNA [2] by agreeing with the nuDNA data that *Platysternon *is a testudinoid. The fact that our large mt alignment results in a phylogenetic hypothesis that is congruent with the nuDNA rather than the analyses based on small (< 5 kb) mt sequences highlights the utility of generating large mt sequences for higher-level systematics [11]. Because independent genetic markers (mtDNA and nuDNA) support testudinoid affinities, and there is no strong morphological argument for chelydrid affinities [10], the continued recognition of *Platysternon *as a chelydrid is no longer tenable.

The paleontological record is consistent with the "*Platysternon *as a testudinoid" hypothesis. The oldest fossil referred to the stem lineage of *Platysternon *(the Platysternidae), are from the Paleogene of Asia (55–60 mya) [12,13], at about the same time we find the oldest testudinoids [14,15]. Chelydrids, on the other hand, are significantly more ancient, extending back into the middle Cretaceous (~90 mya) [16]. Consequently, the recognition of *Platysternon *as a testudinoid alleviates a major temporal disparity of ~30 million years. Despite this apparent congruence, it is important to realize that the reported fossil record of platysternids is poor and in need of review and confirmation [17]. The described material from Asia is based largely on fragmentary specimens that have not been subjected to rigorous phylogenetic analysis [12,13,18-21]. Meanwhile, potentially relevant fossil specimens of possible platysternids in Europe [22] and North America ("Emydid C" [14]) have been mentioned in the literature, but have not been adequately described. The possibility of early platysternids in North America is especially intriguing because our study supports a sister relationship to *Chrysemys*, our representative of the largely North American clade Emydidae. However, until more specimens are brought to light, the paleontological perspective on platysternid origins remains highly speculative.

### Genomic features of *Platysternon *mtDNA

**Figure 5:**
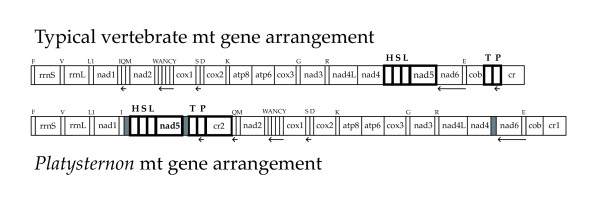
**Mt genomic features of *Platysternon***. Typical arrangement of vertebrate mitochondrial genes including a single control region compared to that of *Platysternon*. All genes are transcribed from left to right except where indicated by arrows. The genes that are rearranged in *Platysternon *are indicated in bold while the duplicated control regions are designated by numerals (cr1, cr2). The grey boxes in the *Platysternon *genome represent non-coding regions (perhaps degraded duplicated copies of genes) that are not present in typical vertebrate mt genomes. This figure illustrates how sequences from two non-adjacent regions were inserted between *trnI *and *trnQ*.

The mtDNAs of vertebrates almost universally contain the same set of 37 genes plus a large, non-coding portion commonly called the "control region" because it contains signals that regulate transcription and replication [23]. Gene rearrangements are not unheard of, but are very uncommon. The mitochondrial genome of *Platysternon *is unusual by having large-scale gene rearrangements and a duplication of the control region (Fig. 5), the two copies of which share 808 nucleotides of identical sequence, and beyond which have no apparent sequence similarity. One of these non-coding regions (1,134 bp) occupies the typical position of the control region (cr) and so we call this "cr1" and the other (1,140 bp) we call "cr2." The ~1,100 bp paralogs have 808 identical positions in the middle that are flanked on either side by polymorphic sequences.

One protein coding gene (*nad5*) and five tRNA genes are in derived positions. These have transposed from two portions of the genome (*trnH*, *trnS*, *trnL*, *nad5 *and *trnT*, *trnP*, cr) that are ancestrally near to one another, but separated by *nad6*, *trnE*, *cob *(Fig. 5). In *Platysternon*, both of these regions are inserted between *trnI *and *trnQ*, are separated by a block of non-coding sequence. This is the first true gene rearrangement reported for a turtle. In the pancake tortoise, *Malacochersus*, the cr and *trnF *are duplicated [24]; however, since the second cr of the pancake tortoise is highly degraded, the two *trnF *are essentially adjacent (i.e., no coding regions are out of sequence). The translocation of the cr and mt genes to between *trnI *and *trnQ *is interesting because this is the same position that contains a duplicated cr and rearranged tRNA genes in another reptile clade, the advanced snakes [25], and because this has been noted otherwise as a rearrangement "hot spot."

The arrangement of the *Platysternon *genome can be modelled by the "duplication-random loss" model [26] whereby a duplication and transposition of part of the genome occurred, then additional rearrangements resulted from the loss of supernumerary genes. Since the transposed genes in *Platysternon *are ancestrally separated by only a block of three genes, it may be that the originally duplicated and transposed region included the entire portion from *trnH *through the cr. This observation bolsters speculation that the non-coding region now found between *nad5 *and *trnT *is the degenerating vestige of what was the duplicated *nad6*, *trnE*, and/or *cob *and, similarly, that the non-coding region between *nad4 *and *nad6 *is the vestige of a copy of *trnH*, *trnS*, *trnL*, and/or *nad5*. The study of recent duplication events demonstrates that when parts of the genome are duplicated, redundant sequences are rapidly lost [27], and cr duplications have been otherwise associated with gene rearrangements [28].

It is unusual that there should be two similar control regions in *Platysternon*, and uncertain whether this indicates a very recent duplication, maintenance by selection, or some error correction mechanism resulting in their evolving in concert. Duplicated control regions have previously been reported for just 11 clades spanning the diversity of Metazoa [25,28-30]. Some experimental data suggest that mt genomes with two crs have a selective advantage in replication over those with one cr [32], but there are clearly cases where one copy of a duplicated cr is degrading [24,28,33].

The maintenance of duplicated sequences is not restricted to crs. A recent study reported seven instances from diverse metazoans, in which reported sequences of coding regions were duplicated [24]. To this we can add the duplication of *trnK *in the reptile *Sphenodon *[29]. Whether all of these duplications represent cases of stable functional redundancy in coding regions or merely result from recent duplications and have not degraded into pseudogenes remains to be tested.

## Conclusion

*Platysternon *is not related to chelydrids, but is instead a member of the Testudinoidea, the group that includes pond turtles and tortoises. Testudinoids diversified rapidly in Asia and North America during the Paleogene (50–60 mya) [14,15]. Additional taxon sampling will help establish the phylogeny for extant testudinoids, including whether *Platysternon *is actually more closely related to emydids or testugurians. However, the best understanding of the timing and geography of this radiation will require the additional description and analysis of important, but neglected, fossil specimens.

The features of the *Platysternon *mitochondrial genome expand our knowledge of variation within vertebrate mitochondrial genomes, adding a new case of duplicated control regions. Moreover, the unusual mt genome of *Platysternon *and the pancake tortoise (*Malacochersus *[24]) are good examples of how additional sequencing of turtle mt genomes can improve our knowledge of mitochondrial variation and evolution. At the time of this writing, just ~6% of turtle diversity (18 of ~300 species) have large (> 5 kb) mt sequences reported (16 of these are complete).

## Methods

### Laboratory protocols

Our new sequences are derived from three museum specimens: 1) *Platysternon megacephalum *(MVZ 230486) from Hainan Island, China; 2) *Chelydra serpentina *(MVZ 137436) from North Carolina, USA; 3) *Kinosternon flavescens *(MVZ 164999) from Texas, USA. Genomic DNA was extracted from frozen liver using the Qiagen QIAamp tissue kit. Amplification of genomic DNA was conducted using rTth long PCR enzyme (Applied Biosystems) with a denaturation at 94°C for 15 sec, annealing at 46–50°C for 20 sec, and extension at 68°C for 60 sec for a total of 38 cycles, followed by an additional extension at 72° for 12 min.

The following primers were used (listed 5' to 3'): A) TestGenPhe.f: AAAGCGTGGCATTGAAGCTG; B) 12Sa: AAACTGGGATTAGATACCCCACT; C) 16sf.2: TACGACCTCGATGTTGSATCAGG; D) TestGenCo3.f: GCTGCTTGATAYTGACACTTYGT; E) Nad4.f5: TGACTACCAAAAGCCCACGTAGA; F) 16S.r10: TCCAACATCGAGGTCGTAAACC; G) Met.r7: GCTATGGGCCCAAAAGCTT; H) Nad4.r6: TCTACGTGGGCTTTTGGTAGTCA; I) Leu.r1: TTTTACTTGGAGTTGCACCA; J) Cb.r24: CTCAGAATGATATTTGTCCTCARGG. The following primer pairs were used for each species: *K. flavescens *(B-I), *C. serpentina *(A-G, C-H, D-J), *P. megacephalum *(A-G, C-H, D-J, E-F).

Amplification products were sheared randomly into fragments of approximately 1.5 kb by repeated passage through a narrow aperture using a Hydroshear device. After end-repair, the sheared DNA was gel purified, ligated into a plasmid vector, and then transformed into bacterial cells to construct a library of random fragments. Automated colony pickers introduced single clones into bacterial broth with 10% glycerol in 384-well format. We sequenced 96 or 192 clones per amplification for 192–576 clones per species (192 for *K. flavescens*, 384 for *C. serpentina*, 576 for *P. megacephalum*). These plasmid clones were processed robotically for rolling circle amplification [34,35]. Sequencing reactions and reaction cleanup were done using SPRI [36]. Sequences were determined using ABI3730xl DNA sequencers and then were assembled based on overlap to form deep contigs 5X->50X).

### Phylogenetic analyses and hypothesis testing

The three new DNA sequences (*Platysternon *[GenBank# = DQ_256377, 19,043 bp complete mt genome], *Chelydra *[DQ_256378, 14,567 bp sequence from *rrnS *to *cob *position 415], *Kinosternon *[DQ_256379, 7,288 bp sequence from *trnL*(taa) to *trnR*]) were aligned manually with those from nine other species from GenBank (*Chelonia *[NC_000886], *Chrysemys *[NC_002073], *Dogania *[NC_002780], *Geochelone *[DQ_080041], *Manouria *[DQ_080040], *Mauremys*/"*Chinemys*" [NC_006082], *Pelodiscus *[NC_006132], *Pelomedusa *[NC_001947], *Testudo *[DQ_080049]). With the exception of *Platysternon*, no noteworthy, unusual genomic features were found in the new sequences. However, we did note that *Kinosternon *lacks the "extra" nucleotide that causes a translational frameshift in *nad3 *in all other turtles where known [24,37].

For our alignment, protein-coding genes were constrained to align by codon and tRNA-coding genes were constrained to align by regions of potential secondary structure [38]. We excluded highly-variable regions that were difficult to align including the control region, 225 positions from other non-coding regions, 79 positions of *rrnS*, and 317 positions of *rrnL*. A total of 170 positions were excluded from the alignment of tRNA genes: the D-loop region was excluded from *trnH*, *trnS*, *trnL*(taa); and both the D- and T-loop regions were excluded from *trnF*, *trnV*, *trnI*, *trnW*, *trnK*, *trnR*, *trnT*, and *trnP*. We excluded a total of 151 positions from the protein coding gene *nad5*. The final alignment contains 15,289 positions and provides 4,901 parsimony informative characters.

We used maximum parsimony (MP) [39], maximum likelihood (ML) [40], and Bayesian inference (BI) [41] phylogenetic methods to infer phylogenetic trees. We conducted the MP and ML analyses with PAUP* 4.0b10 [42] and BI analyses with MrBayes 3.1.1 [43,44]. We executed MP analyses with the branch and bound search option, which guarantees an exact solution. To assess nodal support for the MP analysis, we used the bootstrap resampling method [45] employing 1000 pseudoreplicates of heuristic searches using TBR branch swapping and 100 random sequence additions pseudoreplication in PAUP*. We also obtained decay indices (="branch support") [46] for all nodes.

To determine the most appropriate model of DNA substitution for reconstructing turtle relationships under ML, we evaluated the fit of various models of molecular evolution to our data via the Akaike Information Criterion (AIC) [47] with the program Modeltest 3.06 [48]. We performed ML analyses under the optimal model (GTR + I + G) with the heuristic search algorithm using TBR branch swapping with 10 random sequence additions, simultaneously estimating parameter values (with 10 Γ rate categories) and tree topology (i.e., no initial parameter estimates or starting tree). We then successively re-estimated parameter values and searched for trees until we obtained a stable topology and ML score [49].

We also performed ML-based BI analyses to search for additional tree topologies. Because MrBayes can perform mixed model phylogenetic analyses using different models of evolution [44] we assessed the best fit model of evolution for each mtDNA gene via the AIC with the program MrModeltest 2.1 [50]. However, to avoid over-parameterization, we combined mitochondrial loci into the same data partition if they belonged to the same functional type (either rRNA, tRNA, or protein coding DNA) and conformed to the same model of evolution. This resulted in 12 partitions with the following models: (1) *rrnL*, *rrnS*= GTR+G; (2) *atp6, cox1*, *cox2*, *cox3, nad1*, *nad2*, *nad3*, *nad4*, *nad5 *= GTR+I+G; (3) *cob*, *nad6 *= GTR+G; (4) *atp8 *= HKY+I+G; (5) *nad4L *= HKY+G; (6) *trnA*, *trnD*, *trnG*, *trnQ*, *trnR *= GTR+G; (7) *trnE*, *trnL*(nag) = GTR+I; (8) *trnF *= SYM+G; (9) *trnM *= HKY+I+G; (10) *trnC*, *trnK*, *trnN*, *trnS*(nga), *trnT*, *trnV*, *trnY *= HKY+G; (11) *trnH*, *trnP*, *trnS*(nct) = HKY+I; (12) *trnI*, *trnL*(taa), *trnW *= K80+G.

We then performed mixed-model BI tree searches, allowing separate parameter estimates under the chosen models of DNA substitution for each data partition. We did not specify nucleotide substitution model parameters or a topology *a priori*. We ran BI analyses for 3 × 10^6 ^generations using the default temperature (0.2) with four Markov chains per generation, sampling trees every 100 generations. We then computed a 50% majority rule consensus tree after excluding those trees sampled prior to the stable equilibrium (after the first 1 × 10^5 ^generations). Nodal support is given by the frequency of the recovered clade, which corresponds to the posterior probability of that clade under the assumed models of sequence evolution [43,51].

We assessed the congruence between our hypothesized placement of *Platysternon *and those proposed by other molecular genetic analyses using constraint searches and subsequent topology tests in PAUP*. First, we constrained the MP and ML searches to retain only those trees with a *Platysternon *+ *Chelydra *clade, consistent with previous mtDNA analyses [1]. Second, we constrained the MP and ML searches to retain only those trees with a *Platysternon *+ Testuguria clade, consistent with a previous nuDNA analysis [3]. We then compared the constrained and unconstrained MP estimates of turtle phylogeny using a two-tailed Wilcoxon signed-ranks test [52], and compared the constrained and unconstrained ML phylogenies using a one-tailed multiple-comparisons likelihood ratio test [53] with 1000 RELL bootstrap pseudoreplicates.

Finally, we also performed phylogenetic analyses of a data matrix that combined our mtDNA data with nuDNA from two relevant studies [2,3]. The combined analyses were performed using the same parameters used for the mtDNA analyses given above and with the models for the nuDNA specified in those other studies [2,3]. Because there is non-overlapping taxonomic coverage between the three studies (ours and the two nuclear studies) we had to use Operational Taxonomic Units (OTUs) that had data from more than one species. These "chimeras" are a major problem in turtle systematics, especially in paleontological studies where the inclusion of broadly paraphyletic OTUs is a recurring phenomenon [10]. We tried to avoid this problem by combining nuDNA and mtDNA sequences from only the most closely related taxa to ensure that our OTUs would be monophyletic with respect to one another. The one exception is the trionychids. In that case we combined the RAG-1 sequence for *Apalone *with the large mt sequence from *Pelodiscus *(no U17 snoRNA data is available for any trionychid). This combination was arbitrary since *Apalone *is just as closely related to *Dogania *as *Pelodiscus*, but this should not impact our results since all studies agree on the phylogentic position of these taxa within Cryptodira. The following list gives the OTU name used in Figure 5 followed by the accession numbers for the nuDNA sequences used (EMBL number for U17 snoRNA, GenBank number RAG-1): *Pelomedusa *(AJ306565, AY687922), Trionychidae [*Dogania *(no nuDNA), *Pelodiscus *(no U17 snoRNA, AY687901 from *Apalone*), the analyses were run with two separate trionychid OTUs and they were collapsed into a single terminal in Figure 5], Kinosternidae (AJ306562, AY687911 from *Sternotherus*), *Chelydra *(AJ306559, AY687906), *Chelonia *(AJ493419, AY687907), Emydidae [mtDNA from *Chrysemys*, nuDNA from *Trachemys *(AJ306564, AY687915)], *Platysternon *(AJ493418, AY687905), Geoemydidae [mtDNA and RAG-1 from *Mauremys *(AY687914), U17 snoRNA from *Cuora *(AJ493422)], *Manouria *(no nuDNA), *Geochelone *(AJ306561, AY687912), *Testudo *(AJ306563, no RAG-1).

### Phylogenetic taxonomy

Most of the suprageneric clade names used in this study are based on a recent review of phylogenetic nomenclature for turtles [54]. We follow all of the protocols of that study with the exception of italicizing phylogenetically-defined clade names. Although most of the relevant phylogenetic definitions can be found in that study, a few names require additional discussion. For example, the first worker to hypothesize a close affinity of *Platysternon *and testudinoids [6] also coined the name Cryptoderinea to accommodate this grouping. Cryptoderinea has been phylogenetically codified, but can only be considered valid if *Platysternon *is sister to Testudinoidea [54]. If *Platysternon *is nested within Testudinoidea, as proposed here and in other genetic studies [2,3], then *Playsternon *should be considered a testudinoid and the name Cryptoderinea should not be used [54].

Secondly, the phylogenetically-defined name Bataguridae was proposed for the testudinoid clade that include most Asian hard-shelled turtles [54]. However, according to a strict application of the rules of the International Congress of Zoological Nomenclature, there is an argument for the use of the name Geoemydidae for the same clade. In order to foster consensus during the transition from Linnaean taxonomy [55] to PhyloCode [56], we use the name Geoemydidae for this group.

Finally, despite the fact that a previous study [1] had proposed the name "Testudinoidae" for the clade that includes geoemydids and testudinids, the phylogenetic system used here [54] recommended using a new name, Testuguria. Testuguria was coined because Testudin**oi**d**ae**was deemed too phonetically similar to clade names of the next higher and lower levels (Testudin**oi**dea and Testudinid**ae**respectively). Although not explicitly listed as an objective synonym of Testuguria, Testudinoidae was given in the list of *Testudo *derivatives as an example of what kind of names to avoid. It is important to note that priority can not be invoked because, at the time of this writing, there is no official starting date for the validity of phylogenetically defined definitions. When the time comes to codify these names there will have to be a discussion as to which name (Testuguria or Testudinoidae) should be used. We strongly recommend the use of Testuguria for the reasons given above.

## Authors' contributions

JFP participated in the design of the study, performed all of the laboratory work, acquired and interpreted the sequence data, annotated and aligned the data, assisted the phylogenetic analyses, and coordinated the drafting of the paper with the other authors; CRF participated in the drafting of the paper performed the bulk of the phylogenetic analyses; JLB participated in the design of the study and the drafting of the paper. All authors read and approved the final manuscript.
